# Dynamical analysis of a damped harmonic forced duffing oscillator with time delay

**DOI:** 10.1038/s41598-023-33461-z

**Published:** 2023-04-20

**Authors:** Galal M. Moatimid, T. S. Amer, W. S. Amer

**Affiliations:** 1 Mathematics Department, Faculty of Education, Ain Shams University, Cairo, Egypt; 2 Mathematics Department, Faculty of Science, Tanta University, Tanta, 527 Egypt; 3 Mathematics and Computer Science Department, Faculty of Science, Menoufia University, Shebin El-Kom, 32511 Egypt

**Keywords:** Applied mathematics, Mathematics and computing, Physics

## Abstract

This paper is concerned with a time-delayed controller of a damped nonlinear excited Duffing oscillator (DO). Since time-delayed techniques have recently been the focus of numerous studies, the topic of this investigation is quite contemporary. Therefore, time delays of position and velocity are utilized to reduce the nonlinear oscillation of the model under consideration. A much supplementary precise approximate solution is achieved using an advanced Homotopy perturbation method (HPM). The temporal variation of this solution is graphed for different amounts of the employed factors. The organization of the model is verified through a comparison between the plots of the estimated solution and the numerical one which is obtained utilizing the fourth order Runge–Kutta technique (RK4). The outcomes show that the improved HPM is appropriate for a variety of damped nonlinear oscillators since it minimizes the error of the solution while increasing the validation variety. Furthermore, it presents a potential model that deals with a diversity of nonlinear problems. The multiple scales homotopy technique is used to achieve an estimated formula for the suggested time-delayed structure. The controlling nonlinear algebraic equation for the amplitude oscillation at the steady state is gained. The effectiveness of the proposed controller, the time delays impact, controller gains, and feedback gains have been investigated. The realized outcomes show that the controller performance is influenced by the total of the product of the control and feedback gains, in addition to the time delays in the control loop. The analytical and numerical calculations reveal that for certain amounts of the control and feedback signal improvement, the suggested controller could completely reduce the system vibrations. The obtained outcomes are considered novel, in which the used methods are applied on the DO with time-delay. The increase of the time delay parameter leads to a stable case for the DO, which is in harmony with the influence of this parameter. This drawn curves show that the system reaches a stable fixed point which assert the presented discussion.

## Introduction

In reality, oscillation is a regular occurrence. It has the potential to both cause damage and yield benefits in life. Whenever the oscillation reaches a particular threshold, it can cause damage to human and animal health, keeping the human body discomfort and preventing technology from working regularly. Vibrations have advantages where they can be transformed into other forms such as types of energy, create lovely music, send out signals, and so on. This will have a significant impact on the advancement of human living standards. Consequently, vibration controllers and employment have been developed as fundamental technology and challenge in the mechanical development and production of current high-tech machinery. The relevance of time-delayed differential equations has led to the development of a substantial area of nonlinear analysis with extensive applications in biology, economics, physics, nuclear reactor, chemistry, and natural science fields. In powerful vibrating controllers, the time delay is necessary and has been previously seen as a spontaneous feature that can destabilize some stable systems. Therefore, differential equations with time-delayed influences have attracted much interest in current history since they have been demonstrated to be helpful resources^1–3^. Nonetheless, time-delay has been used lately in a number of research papers as a controlling factor that can be used to develop the regulator effectiveness.

Actually, numerous physical processes have been represented by employing nonlinear ordinary differential equations in the previous decades. The DO is just one of these equations that have attracted great attention because of its conventional uses in various areas. Several researchers are motivated by this nonlinear differential equation because it reflects the normalization approach in our real world, based on its unique oscillating and chaotic environment^4–6^. The DO arises when a body moves under the influence of nonlinear spring strength, linear stickiness dampening, and periodic powers^7^. It could be used to establish mechanical experiences and significant changes by periodical exterior strength. An effort to explore several DO applications and propose an alternate mathematical model to approximate them was made^8^. In this approach, the power series was implemented as the beginning function through the integration brought about by a one-step interval. With regard to calculations of circuit analysis, the DO is supplementary and particularly difficult to deal with. Therefore, Chen et al.^9^ scrutinized the circuits from the standpoint of vibrating sciences and energetics available to support the investigation of their properties. As a result of this work, users will be able to comprehend the mechanics and properties of DO in a much more straightforward way. The influence of an increased simulation on significant stationary-state formulations and bi-stability was investigated^10^ in a delayed DO. The fundamental equation of the slow dynamical oscillator and the related flow was obtained using the honest partitioning of motion approach and the multiple scales method^11^. Numerical computations have validated the quantitative outcomes of this study. This method was used in many works^12–15^ to attain the estimated formulas of various vibrational models. The stability of the parametric DO was investigated^16^. The decelerated factor and the cubic stiffening factor were shown to have a destabilizing effect. Fundamental and parametric harmonics have stabilizing effects. The cubic-quintic nonlinear term and the externally stimulated impulse were explored in a combined Rayleigh-Van der Pol-Duffing oscillator^17^. To provide an approximate solution, the Poincare'–Lindstedt methodology was modified^11^. A comparison of the estimated approach and RK4 has revealed significant compatibility.

Differential equations are widely utilized in computational chemistry in addition to industrial biomechanics in developmental stages. Resolutions towards such equations have been required to assess and enhance reactions in a mathematical conception. Computing an accurate solution is challenged in the majority of situations. To understand such difficulties, a variety of mathematical approaches has been modeled. Therefore, simplifications and computational methodologies are widely employed since most equations may not have an accurate solution. Subsequently, many classical perturbation methods were recognized. Unfortunately, all of these methods necessitate the existence of a small parameter (SP) in the handheld equation. The establishment of a synthetic SP is frequently motivated by the lack of a meaningful physical application of a zone to be too limited. In a few words, the SP may be applied in a variety of ways. So, the supplementary critical question arises. Is it imperative to attain information immediately? It may be done by using SP or using a synthetic one. In this regard, it is preferable to talk properly about “methodologies specialized in parameter improvement instead of merely the SP. Really, there is no distinction between real and artificial SP from this viewpoint. Nevertheless, the expression manufactured SP should be used again as is customary. It is worth mentioning that the presence of SP has been suggested in a diversity of mathematical fields. The Chinese Mathematician Prof. He^18–20^ created the HPM in response to the constraints of the classical perturbation methods. As an essential concept, the SP has no significance on the HPM. In accordance with the Homotopy equation, the given problem may be constructed into two linear and nonlinear parts and divided by the parameter $\rho \in \left[ {0,1} \right]$. It has been demonstrated that it can be used to analyze highly nonlinear vibrational issues and create a multiplicity of real-world engineering challenges. The fundamental equations of exceedingly nonlinear excitations of orthotropic structural components are deduced using the Von Karman huge defection hypothesis and the Galerkin method to achieve a precise and effective alternative approach to the nonlinear vibrational problem of the membrane^21^. Using HPM, an analytical solution to the problem of free vibration of rectangular plates with convoluted border circumstances in a plane was suggested^22^. The HPM was used to introduce a solution to a time-delayed differential equation and was followed by numerical illustrations^23^. The outcomes demonstrated that the suggested strategy is both successful and straightforward to implement. For an active vibration control of a beam on an elastic base, the HPM was used^24^. The methodology was used to load an axially loaded elastic basis with one fixed end, while the other was simply maintained. In comparison to other interpolating techniques, it has been shown that HPM provides a highly accurate solution, in association with the other techniques, for a specific problem.

The concept of control was widely incorporated into the modeling of autonomous engines or physiological systems, even though the feedback was required to maintain a stable state. But a lot of this feedback required a short amount of time to analyze the data. It was common practice to describe this occurrence by formulating a delay differential equation (DDE), in which the evolution of a dependent variable at a time $t$ depends on its value at a time $(t - \tau )$. Unfortunately, it was a very difficult mathematical problem to solve a DDE. In the last years, there has been a significant increase in processing power, which has reignited interest in DDEs^25^. The Adomian polynomials now have a new formula^26^. A class of nonlinear differential equations was studied using this novel formula. The Adomian solution utilizing this new formula converges more quickly, according to a numerical experiment. Almost all scientific disciplines, including mathematics, physics, engineering, biology, neuroscience, physiology, economics, and many more, are connected by the topic of dynamic systems with time delay, which was an active area of research. Together, contributions from theoretical and experimental groups were highlighted, stressing a wide range of applications^27^. There were reports on systematic bifurcation studies of mode-locked laser systems, higher-order locking phenomena between delay and laser oscillation period, and management of cavity solitons as light spots in spatially extended systems. Both experimental and analytical methods were used to examine the nonlinear recovery of reverse-biased waveguide absorbers based on quantum dots^28^. The recovery dynamics were demonstrated to consist of a quick initial layer and comparatively moderate decay. The carrier capture, escape, and Pauli blocking processes were taken into consideration in a rate equation model of a quantum dot semiconductor optical amplifier^29^. It examined potential distinctions between Auger processes that are prominent for recovery and phonon-assisted processes. Experimental, numerical, and analytical research were done to determine how a nonlinear optical oscillator will react to delayed broadband bandpass filtering feedback^30^.

The examination of the DO, in light of the previous advantages, has potential requests in a wide range of physical and technological phenomena. Therefore, continuing employment studies on the stability analysis of the parametric DO have an extensive variety of applications in science and engineering. A second-order ordinary differential equation with a cubic-quintic nonlinearity is represented in this equation. To establish a better approximate solution, the HPM is joined with the Laplace transform, and the idea of expanding frequency will be utilized. A comparison with RK4 is made to verify the prototypical association. Consequently, the existing work mainly discussed DO and studied its excitation with location and velocity delays in the following way:

1 $$\ddot{y} + \omega^{2} y + 2\mu \dot{y} + \alpha y^{3} + \beta y^{5} = F\cos \Omega t + 2\gamma y(t - \tau ) + 2\lambda \dot{y}(t - \tau ),$$

where all of the used constants in Eq. (1) are previously provided.

To crystallize the implementation of our inquiry, the rest of the paper will be outlined along with the following Sections: an improved approximate solution to the considered problem using the improved HPM is explained in Section "Improved HPM". Section "Discussion of the HPM outcomes" is devoted to the discussion of the previous outcomes. The mathematical procedure of the nonlinear stability analysis is introduced, and the primary resonance is established in Section "Mathematical Formulation of nonlinear analysis". The frequency response on numerical validations is discussed in depth in Section "The frequency–response curves and numerical validations". Additionally, its subsections examine the controlled system with/without the time delay as well as the uncontrolled system. Finally, the main findings of the current study are summarized in “Conclusion” Section.

It is easier to imagine the initial circumstances as follows:

2 $$y(0) = 0,\,\,\,\,\,\,\dot{y}(0) = 1.$$

The HPM technology is constructed mostly on the underlying Homotopy equation:

3 $$\ddot{y} + \omega^{2} y = \rho [ - 2\mu \dot{y} - \alpha y^{3} - \beta y^{5} + F\cos \Omega t + 2\gamma y(t - \tau ) + 2\lambda \dot{y}(t - \tau )];\,\,\rho \in \left[ {0,1} \right].$$

## Improved HPM

The standard method could be changed to respond accurately over time. Such a skillfully adapted HPM could very well reveal the previously underappreciated importance of the time delay parameter. Nevertheless, it also addresses the problem by using an exponential delay to offer a corresponding characteristic across the decay constriction. One can get a constrained solution over time, and that is the topic of this essay. To explore the effects of the delay parameter, we might evaluate Homotopy Eq. (3) using the new expansion that departs from the traditional one. In conclusion, the time-dependent amount can indeed be increased in the way described^31^:

4 $$y(t,\rho ) = e^{ - \rho \tau \;t} \{ y_{0} (t) + \rho \;y_{1} (t) + \rho^{2} y_{2} (t) + \ldots \} ,$$

where $y_{j} \,\,(j = 0,1,2)$ are the stages of $y$. The recognized natural frequency can be enlarged as follows using the methodology of the previously understood investigation^16^:

5 $$\varpi^{2} = \omega^{2} + \sum\limits_{i = 1}^{n} {\rho^{i} } \omega_{i} .$$

The developing formula that is provided in Eq. (4) is inserted into the Homotopy equation, in light of the starting conditions as given in Eq. (2), to generate an improved approximate solution of Eq. (3). As an outcome, the analytic mathematical formulation of the zero-order problem is specifically defined as follows:

6 $$y_{0} (t) = \frac{1}{\varpi }\sin \,\varpi t.$$

The first problem of the Homotopy Eq. (3) can be described as:

7 $$\ddot{y}_{1} + \varpi^{2} y_{1} = F\cos \Omega t + t\tau \varpi^{2} y_{0} + \omega_{1} y_{0} - \alpha y_{0}^{3} - \beta y_{0}^{5} + 2\gamma y_{0} (t - \tau ) - 2\mu \dot{y}_{0} + 2\tau \dot{y}_{0} + 2\lambda \dot{y}_{0} (t - \tau ) + t\tau \ddot{y}_{0} .$$

The regularly homogeneous improvement requires the removal of the secular associations when Eq. (6) is involved in Eq. (7). In general, the coefficients of the circular functions $\sin \varpi t$ and $\cos \varpi t$ reflect the behavior of the fundamental regular situations. Therefore, the uniform valid approximate solution needs the following restrictions:

8 $$\lambda \varpi \cos \varpi \tau + \varpi (\tau - \mu ) - \gamma \sin \varpi \tau = 0,$$

and

9 $$8\varpi^{4} (2\gamma \cos \varpi \tau + 2\lambda \varpi \sin \varpi \tau + \omega_{1} ) - 5\beta - 6\alpha \varpi^{2} = 0.$$

The solvability criteria are specified by Eqs. (8) and (9), respectively.

Our aim, away from the presence of secular terms, is to obtain a consistently reasonable solution for the first-order stage $y_{1}$. At this point, the first-order Eq. (7) then becomes

10 $$\ddot{y}_{1} + \varpi^{2} y_{1} = \frac{1}{{16\varpi^{5} }}[16F\varpi^{5} \cos \Omega t + (5\beta + 4\alpha \varpi^{2} )\sin 3\varpi t - \beta \sin 5\varpi t].$$

Taking Laplace transforms for both sides of Eq. (10), we attain the following solution

11 $$y_{1} = \frac{1}{{16\varpi^{5} }}\left\{ {\frac{{16 F \varpi^{5} \cos \Omega t}}{{(\varpi^{2} - \Omega^{2} )}} - \frac{1}{{6\varpi^{2} (\varpi^{2} - \Omega^{2} )}}\left[ {96\varpi^{7} F \cos \varpi t + (9\alpha \varpi^{2} + 10\beta )(\Omega^{2} - \varpi^{2} )} \right]\sin \varpi t - \frac{1}{{8\varpi^{2} }}(5\beta + 4\alpha \varpi^{2})\sin 3\varpi t + \frac{\beta }{{24\varpi^{2} }}\sin 5\varpi t} \right\}.$$

One more, solving Eqs. (8) and (9), we achieve

12 $$\cos \varpi \tau = \frac{{5\beta \gamma + 6\alpha \gamma \varpi^{2} - 8\gamma \varpi^{4} \omega_{1} + 16\lambda \varpi^{6} (\mu - \tau )}}{{16\varpi^{4} (\gamma^{2} + \lambda^{2} \varpi^{2} )}},$$

and

13 $$\sin \varpi \tau = \frac{{5\beta \lambda \varpi + 6\alpha \lambda \varpi^{3} - 8\lambda \varpi^{5} \omega_{1} + 16\gamma \varpi^{5} (\tau - \mu )}}{{16\varpi^{4} (\gamma^{2} + \lambda^{2} \varpi^{2} )}}.$$

To this order of approximation, Eqs. (12) and (13) are combined, keeping in mind $\omega_{1} = \varpi^{2} - \omega^{2}$, to construct the characteristic equation of the expanded nonlinear frequency as:

14 $$\begin{aligned} \varpi^{14} & + \frac{1}{{64\lambda^{2} }}\{ 64[\gamma^{2} + 2\lambda^{2} (2\mu^{2} + 2\tau^{2} - 2\lambda^{2} - 4\mu \tau - \omega^{2} )]\varpi^{12} - 64[2\omega^{2} (\gamma^{2} - \lambda^{2} \omega^{2} ) \\ & + 4\gamma^{2} (2\lambda^{2} - 2\tau^{2} - \mu^{2} ) + \frac{3}{2}\alpha \lambda^{2} + 8\gamma^{2} \mu \tau ]\varpi^{10} - 64[\frac{3}{2}\alpha (\gamma^{2} - \lambda^{2} \omega^{2} ) + 4\gamma^{4} + \frac{5}{4}\beta \lambda^{2} \\ & - \gamma^{2} \omega^{4} ]\varpi^{8} + [80\beta (\lambda^{2} \omega^{2} - \gamma^{2} ) + 4\alpha (9\alpha \lambda^{2} + 28\gamma^{2} \omega^{2} )]\varpi^{6} + 4(20\beta \gamma^{2} \omega^{2} + 15\alpha \beta \lambda^{2} \\ & + 9\gamma^{2} \alpha^{2} )\varpi^{4} + \beta (60\alpha \gamma^{2} + 25\beta \lambda^{2} )\varpi^{2} + 25\beta^{2} \gamma^{2} \} = 0. \\ \end{aligned}$$

To this end, the periodic estimated solution of the prepared principle of movement in Eq. (1) can be represented as follows:

15 $$y = \mathop {\lim }\limits_{\rho \to 1} \,e^{ - \rho t\tau } (y_{0} (t) + \rho y_{1} (t)).$$

## Discussion of the HPM Outcomes

This section is devoted to graphing and analyzing the achieved outcomes by using the improved HPM. To confirm the approach of our procedure, it is desirable to display both the analytical and numerical solutions. A prototype specified selection system that has the following characteristics is used:

$$\begin{gathered} \alpha = 0.25,\,\,\,\,\,\,\beta = 0.5,\,\,\,\,\,\,\,\gamma = 2.0,\,\,\,\,\,\,\,\lambda = 0.8,\,\,\,\,\,\,\tau = 0.5, \hfill \\ \mu = 0.05,\,\,\,\,\,\,\,\omega = 4.5,\,\,\,\,\,\,\,\Omega = 2.0,\,\,\,\,\,\,\,F = 0.1. \hfill \\ \end{gathered}$$

**Figure 1:**
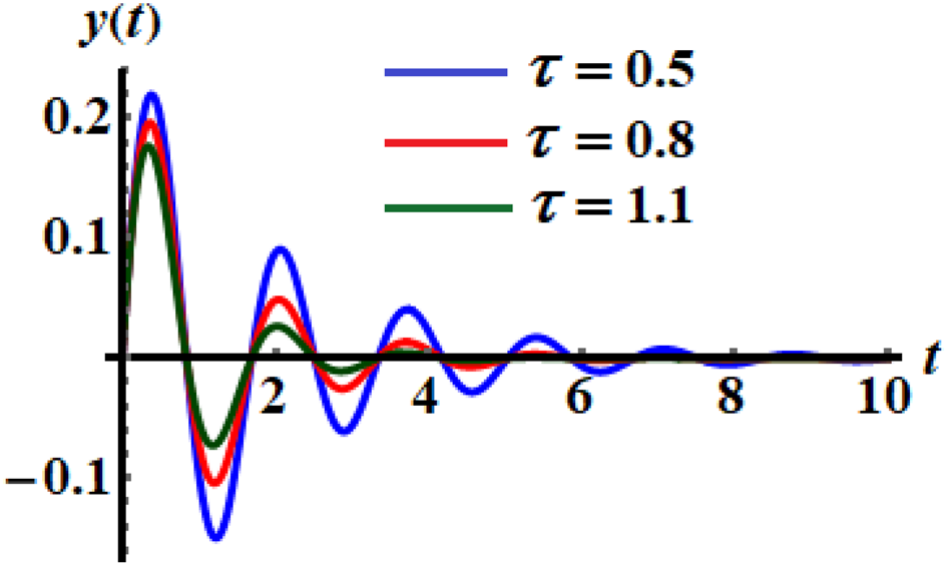
The temporal histories of the AS when $\tau ( = 0.5,0.8,1.1)$.

**Figure 2:**
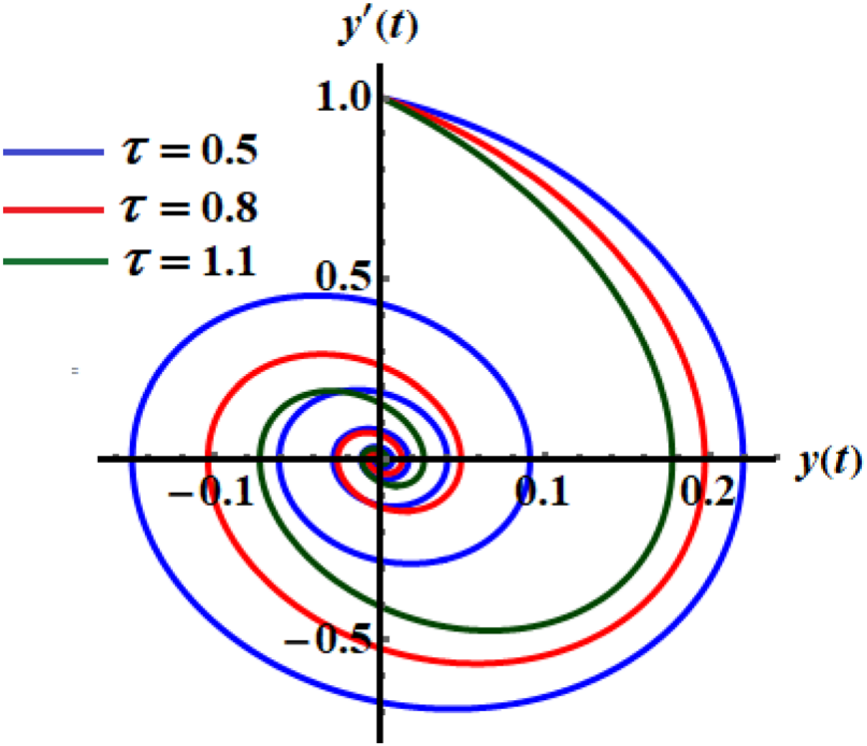
The phase plane curves of the represented curves of Fig. 1.

Therefore, the analytic solution (AS) is graphed in Fig. 1 when the decay parameter has the following different amounts $\tau ( = 0.5,0.8,1.1)$. The subsequent figure is calculated when one of the real roots of Eq. (14) is considered. With the aid of the Mathematica Software, we choose the real reasonable value of the nonlinear expanded frequency as $\varpi = 6.84115$. As shown, the curves of Fig. 1 have an oscillatory decay form till the end of the examined time interval which is consistent with Eq. (15). When $\tau$ increases, the oscillations decay increases, and the curves have a stationary performance at the end of the time interval. The corresponding phase plane diagrams of these curves are drawn in Fig. 2. It is observed that the curves start from one point, which is the initial point, and have spiral forms until the end at another stable point. This signifies that the obtained solutions are stable.

**Figure 3:**
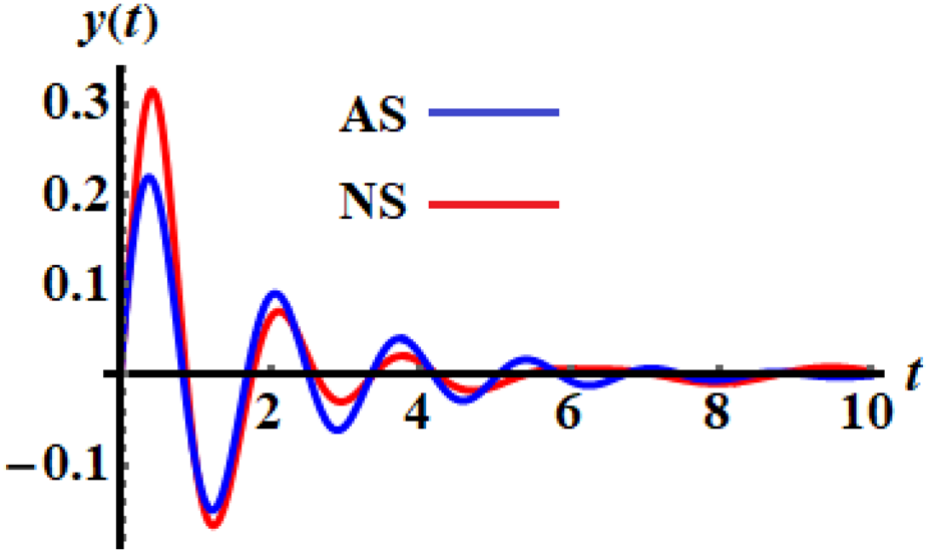
Signifies the connection of the AS and the NS at $\tau = 0.5$.

**Figure 4:**
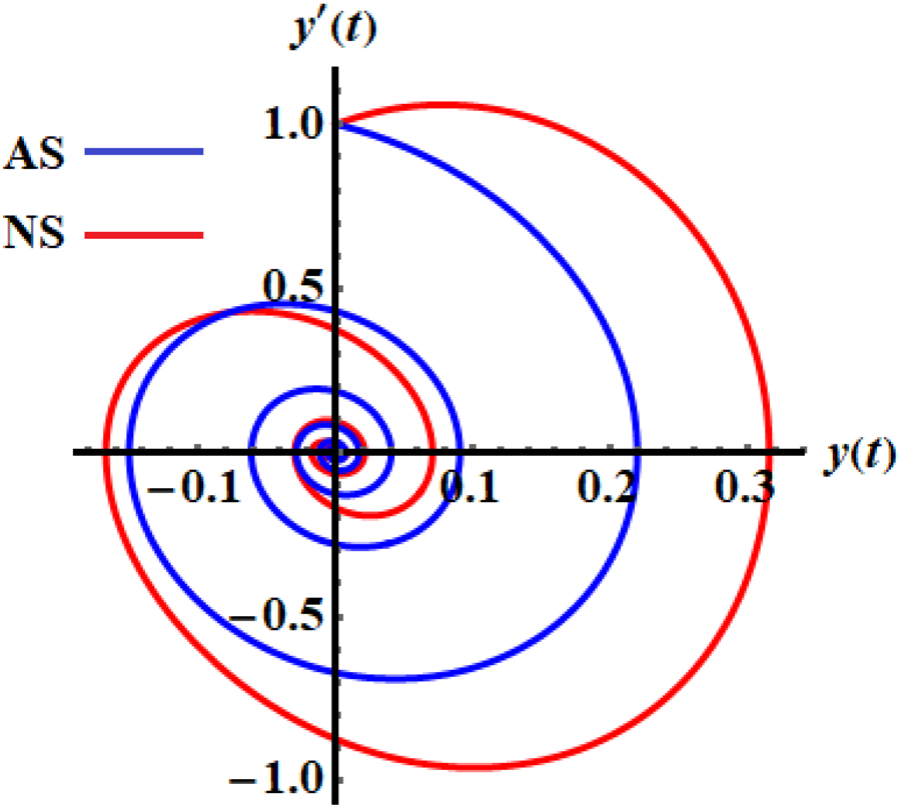
Shows the phase plane diagrams of the curves of Fig. 3.

**Figure 5:**
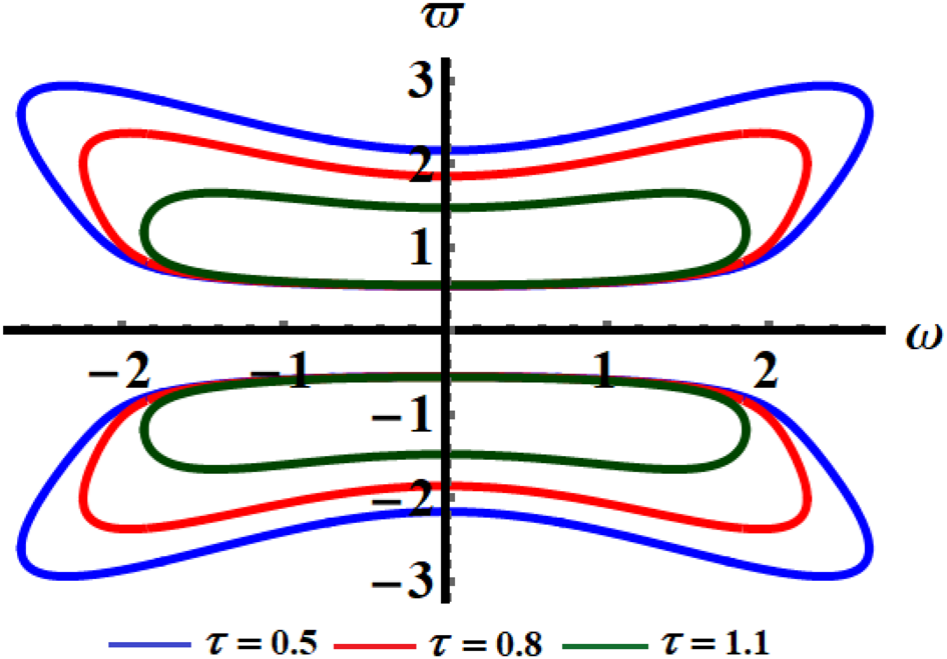
Illustrates the relation between $\omega$ and $\varpi$ at $\tau ( = 0.5,0.8,1.1)$.

The numerical solution (NS) of Eq. (1) is obtained by employing the RK4 and is compared with the AS when the previous data are used at $\tau = 0.5$ as shown in the curves of Fig. 3, while the phase plane curves are drawn in Fig. 4. The difference between them is due to the forms of Eqs. (1) and (15). The curves of Fig. 5 reveal the solution of Eq. (14), when the time delay parameter has various values and interprets the relation between $\omega$ and $\varpi$. This figure includes three symmetric closed curves about the $\omega$ axis when the time dealy parameter has various values, which is consistent with the mathematical form of Eq. (14).

## Mathematical formulation of nonlinear analysis

Throughout this Section, forced and damped vibrations are considered by means of the multiple scale homotopy perturbation method. The solutions will be verified with the numerical imitations. Presume the following hampered structure with exterior excitement.

16 $$\ddot{y} + \omega^{2} y + 2\mu \dot{y}^{2} + \alpha y^{3} + \beta y^{5} = F\cos \Omega t + F_{c} ,$$

where $F_{c}$ is a controller force. Two various control algorithms that involve the linear position and velocity controls are established. Consequently, the controller force can be formulated as:

17 $$F_{c} = 2\gamma y(t - \tau ) + 2\lambda \dot{y}(t - \tau ),$$

where $\gamma$ and $\lambda$ are the linear gains of the position and velocity feedback control.

**Figure 6:**
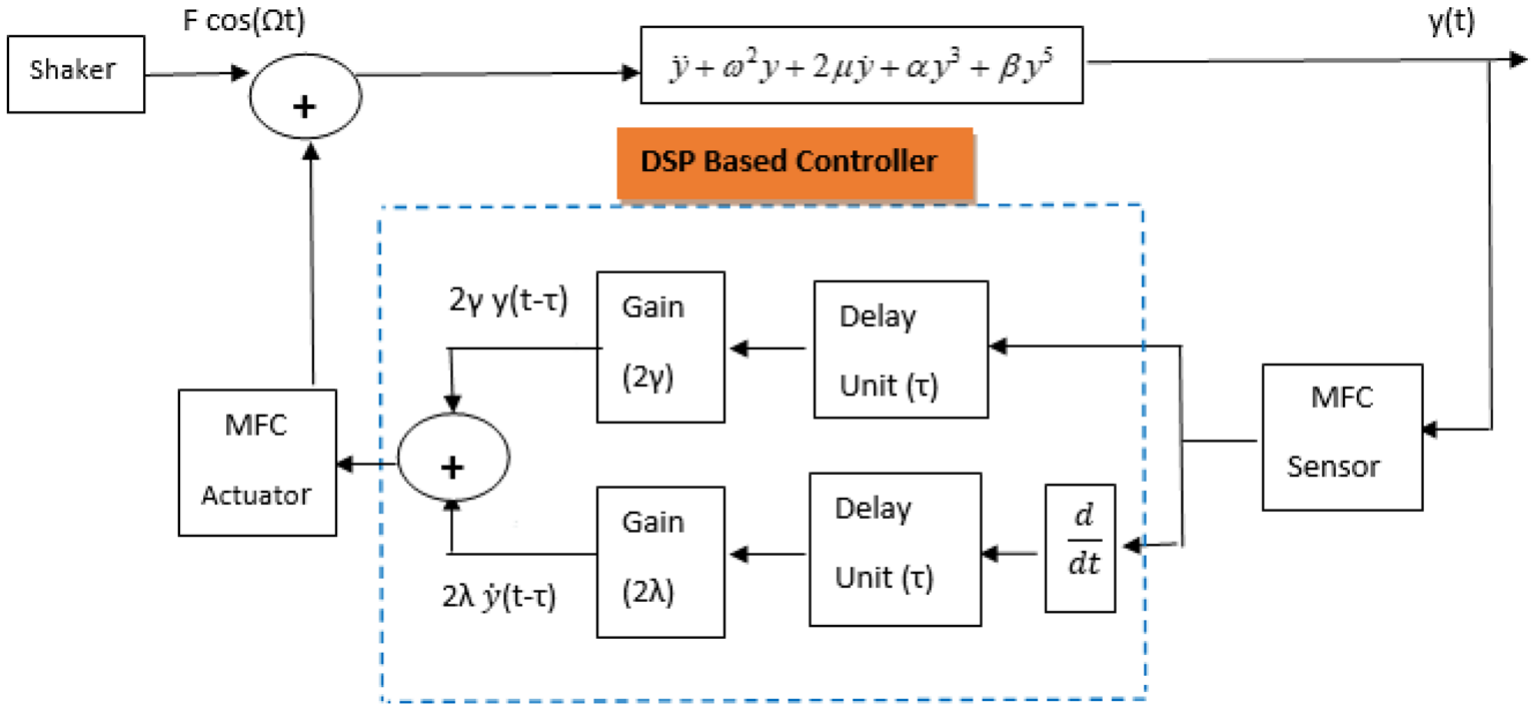
Explores the controlled system block diagram.

The engineering explanation of the analog-to-digital converter (A/D) is shown in Fig. 6. This figure shows how the A/D process can be executed sequentially, where the macro-fiber composite (MFC) sensor measures the oscillations of the system. It follows that the measured signal is fed to a digital signal processor (DSP) that acts as a controller via an A/D. The previous control algorithm performs the required mathematical processing for the acquired signal. The processed signal is then fed back via a power amplifier to the MFC actuator which, in turn, improves the vibrational behavior of the system. Therefore, to compare the efficiencies of the proposed controllers and to report the optimum control factors, a nonlinear examination of the whole structure (i.e., Eqs. (16) and (17) is established. However, to choose the optimal control factors ($\gamma ,\lambda ,$ and $\tau$) that can minimize the oscillation amplitude of the assumed scheme, a scientific examination of the impact of these parameters on the scheme of dynamical performance must be presented by evaluating the scheme of dynamical Eq. (1). Whereas the hampering and exterior excitement with the control parameters are rearranged so that they can balance the impact of nonlinearity on the maximum order. This choice is particularly appropriate for the basic resonances of oscillations.

### Main resonance situation $(\Omega \cong \omega + \sigma )$

As the structure is affected by extreme oscillation amplitude and excitation force at the main primary resonance case, see Nayfeh and Mook^32^, the homotopy equation of the model at hand can be formulated as:

18 $$H(y,\rho ) = L(y) + \rho N(y),\,\,\,\,\rho \in [0,1],\,$$

where $\rho$ is the embedded artificial homotopy parameter, and $L(y)$ and $N(y)$ are the linear and nonlinear portions of the assumed differential equation, correspondingly. They are identified as:

19 $$L(y) = \ddot{y} + \omega^{2} \,y,$$

and

20 $$N(y) = 2\mu \dot{y} + \alpha y^{3} + \beta y^{5} - F\cos \Omega t - 2\gamma y(t - \tau ) - 2\lambda \dot{y}(t - \tau ).$$

Consequently, the homotopy formula can be constructed as:

21 $$H(y,\rho ) = \ddot{y} + \omega^{2} y + \rho [2\mu \dot{y} + \alpha y^{3} + \beta y^{5} - F\cos \Omega t - 2\gamma y(t - \tau ) - 2\lambda \dot{y}(t - \tau )].$$

Without sacrificing generality, two-time scales could be contemplated. Characteristically, the shift may be extended as follows:

22 $$y(t,\rho ) = y_{0} (T_{0} ,T_{1} ) + \rho y_{1} (T_{0} ,T_{1} ) + O(\rho^{2} ),$$

and

23 $$y(t - \tau ,\rho ) = y_{0} (T_{0} - \tau ,T_{1} - \rho \tau ) + \rho y_{1} (T_{0} - \tau ,T_{1} - \rho \tau ) + O(\rho^{2} ),$$

where $T_{0} = t$ and $T_{1} = \rho t$. It follows that the time derivatives $\frac{d}{dt}$ and $\frac{{d^{2} }}{{dt^{2} }}$ can be expressed using the time scales $T_{0}$ and $T_{1}$ as:

24 $$\frac{d}{dt} = D_{0} + \rho D_{1} , \, \,\,\,\,\frac{{d^{2} }}{{dt^{2} }} = D_{0}^{2} + 2\rho D_{0} D_{1} ;\,\,\,\,\,\,D_{j} = \frac{\partial }{{\partial T_{j} }}\,\,\,\,(\,j = 0,\,1).$$

Replacing Eqs. (22) to (24) into Eq. (21), then comparing quantities of similar powers of $\rho$, one gets:

25 $$\rho^{0} :D_{0}^{2} y_{0} + \omega^{2} y_{0} = 0,$$

26 $$\rho :D_{0}^{2} y_{1} + \omega^{2} y_{1} = - 2D_{0} D_{1} y_{0} - 2\mu D_{0} q_{0} - \alpha y_{0}^{3} - \beta y_{0}^{5} + F\cos \Omega T_{0} + 2\gamma y_{0} (T_{0} - \tau ,T_{1} - \rho \tau )\, + 2\lambda D_{0} y_{0} (T_{0} - \tau ,T_{1} - \rho \tau_{1} ).$$

The solution of Eq. (25) could be formulated as:

27 $$y_{0} (T_{0} ,T_{1} ) = A(T_{1} )e^{{i\omega T_{0} }} + cc,$$

where $cc$ signifies the complex conjugate of the previous term and $A$ is an unidentified complex function that can be evaluated later. Substituting Eq. (27) interested in (26), one finds:

28 $$D_{0}^{2} y_{1} + \omega^{2} y_{1} = ( - 2i\omega D_{1} A - 2i\mu \omega A - 3\alpha A^{2} \overline{A} - 10\beta A^{3} \overline{A}^{2} + \frac{F}{2}e^{{i\sigma T_{1} }} + 2\gamma Ae^{ - i\omega \tau } + 2i\omega \lambda Ae^{{^{ - i\omega \tau } }} )e^{{i\omega T_{0} }} - \alpha A^{3} e^{{3i\omega T_{0} }} \, - \beta (A^{5} e^{{5i\omega T_{0} }} + 5A^{4} \overline{A}e^{{3i\omega T_{0} }} ) + cc.$$

Let $\sigma$ denote a detuning parameter that expresses the nearness of the excitement frequency $\Omega$ to the scheme natural frequency $\omega$ such as $(\Omega = \omega + \rho \sigma )$. To attain an unbroken effective expression, the secular terms should be excluded. The removal of these parts needs the termination of the quantities of the functions $e^{{ \pm i\omega T_{0} }}$. Accordingly, one derives the subsequent solvability situation as follows

29 $$- 2i\omega D_{1} A - 2i\mu \omega A - 3\alpha A^{2} \overline{A} - 10\beta A^{3} \overline{A}^{2} + \frac{F}{2}e^{{i\sigma T_{1} }} + 2\gamma Ae^{ - i\omega \tau } + 2i\omega \lambda Ae^{ - i\omega \tau } = 0.$$

Appropriately, the solution of Eq. (28) must be formulated as:

30 $$y_{1} (T_{0} ,T_{1} ) =- \frac{1}{{8\omega^{2} }}\alpha A^{3} (T_{1} )e^{{3i\omega T_{0} }} + \frac{1}{{24\omega^{2} }}\beta A^{5} (T_{1} )e^{{5i\omega T_{0} }} + \frac{5}{{8\omega^{2} }}\beta A^{4} (T_{1} )\overline{A}(T_{1} )e^{{3i\omega T_{0} }} + cc.$$

Multiplying Eq. (29) by $\rho$, and then considering the tendency to unity yield.

31 $$- 2i\omega \frac{dA}{{dt}} + A( - 2i\mu \omega + 2\gamma e^{ - i\omega \tau } + 2i\omega \lambda e^{ - i\omega \tau } ) - 3\alpha A^{2} \overline{A} - 10\beta A^{3} \overline{A}^{2} + \frac{F}{2}e^{i\sigma t} = 0.$$

Equation (31) denotes a first order nonlinear differential equation with a complex coefficient; the solution of the function $A(t)$ can be stated in the following polar form:

32 $$A(t) = \frac{1}{2}a(t)e^{i\psi (t)} ,$$

where $\psi (t)$ and $a(t)$ are two real functions concerning time.

The above functions correspond to both the improved phase-angle and the vibration amplitude of the scheme, correspondingly. Inserting Eq. (32) in Eq. (31) and dividing the real and the imaginary elements, one finds the following amplitude-phase adjustment equations:

33 $$\dot{a} = \frac{1}{\omega }( - a\omega \mu - a\gamma \sin \omega \tau + a\lambda \omega \cos \omega \tau ),$$

and

34 $$\dot{\phi } = \frac{1}{\omega }(\sigma \omega + \gamma \cos \omega \tau + \lambda \omega \sin \omega \tau - \frac{3}{8}\alpha a^{2} - \frac{5}{16}\beta a^{4} ) + \frac{1}{2\omega a}F\cos \,\phi .$$

where $$\phi = \sigma t - \psi .$$

At steady-state vibrations, one gets $\dot{a} = \dot{\phi } = 0$^27^; therefore, Eqs. (33) and (34) become

35 $$\frac{1}{2}F\sin \phi = a\omega \mu + a\gamma \sin \omega \tau - a\lambda \omega \cos \omega \tau ,$$

and

36 $$\frac{1}{2}F\cos \phi = \frac{3}{8}\alpha a^{3} + \frac{5}{16}\beta a^{5} - \sigma \omega a - \gamma a\cos \omega \tau - \lambda a\omega \sin \omega \tau .$$

The combination of Eqs. (35) and (36) yields

37 $$\frac{1}{4}F^{2} = a^{2} \;(\omega \mu + \gamma \sin \omega \tau - \lambda \omega \cos \omega \tau )^{2} + a^{2} \;(\sigma \omega + \gamma \cos \omega \tau - \frac{3}{8}\alpha a^{2} + \lambda \omega \sin \omega \tau - \frac{5}{16}\beta a^{4} )^{2} .$$

In the stability diagram, the amplitude vibration will be graphed against any factor of the scheme like $\sigma ,F,\gamma ,\lambda$ or $\tau$. Using Mathematica software, the following diagrams will be demonstrated soon in the following section. As opposed to that, the linearized stability can be investigated near the fixed points (equilibrium points). Therefore, one can use the following consistent assumptions^33,34^:

38 $$a = a_{10} + a_{11} ,\;\;\;\phi = \phi_{10} + \phi_{11} \; \Rightarrow \;\dot{a} = \dot{a}_{11} ,\;\;\;\dot{\phi } = \dot{\phi }_{11} .$$

Inserting Eq. (38) into Eqs. (33) and (34), and retaining just the linear elements, a little departure from $a_{11}$ and $\phi_{11}$ is obtained. The following standard expression is the outcome of this approach

39 $$\left( \begin{gathered} \dot{a}_{11} \hfill \\ \dot{\phi }_{11} \hfill \\ \end{gathered} \right) = \left( \begin{gathered} \frac{1}{\omega }( - \omega \mu - \gamma \sin \omega \tau + \lambda \omega \cos \omega \tau )\,\,\,\,\,\,\,\,\,\,\,\,\,\,\,\,\,\,\,\,\,\frac{F}{2\omega }\cos \phi_{10} \hfill \\ - (\frac{3}{4\omega }\alpha a + \frac{5}{4\omega }\beta a^{3} + \,\frac{F}{{2\omega a_{10}^{2} }}\cos \phi_{10} )\,\,\,\,\,\,\,\,\,\,\, - \frac{F}{{2\omega a_{10} }}\sin \phi_{10} \hfill \\ \end{gathered} \right)\,\,\left( \begin{gathered} a_{11} \hfill \\ \phi_{11} \hfill \\ \end{gathered} \right).$$

Established on the last square matrix (Jacobian matrix), the stability of Eq. (39) can be investigated by examining the Jacobian matrix eigenvalues.

40 $$\left| \begin{gathered} (s_{1} - \delta )\,\,\,\,\,\,\,\,\,\,\,\,\,\,\,\,\,\,\,\,\,\,\,\,\,\,\,\,\,\,\,\,\,\,\,\,s_{2} \hfill \\ \,\,\,\,\,\,\,\,\,\,\,\,\,\,\,s_{3\,\,\,\,\,\,\,\,\,\,\,\,\,\,\,\,\,\,\,\,\,\,\,\,\,\,\,\,\,\,\,\,\,\,\,\,\,\,\,\,} \,(s_{1} - \delta ) \hfill \\ \end{gathered} \right| = 0,$$

where

41 $$s_{1} = - \mu - \frac{\gamma }{\omega }\sin \omega \tau + \lambda \cos \omega \tau ,$$

42 $$s_{2} = \frac{{a_{10} }}{\omega }(\sigma \omega + \gamma \cos \omega \tau + \lambda \omega \sin \omega \tau - \frac{3}{8}\alpha a_{10}^{2} - \frac{5}{16}\beta a_{10}^{4} ),$$

43 $$s_{3} = - \frac{1}{4\omega }(3\alpha a_{10} + 5\beta a_{10}^{3} ) + \frac{{s_{2} }}{{a_{10}^{2} }}.$$

The accompanying mathematical expression is then applied:

44 $$\delta^{2} - 2s_{1} \delta + s_{1}^{2} - s_{2} s_{3} = 0.$$

Therefore, the following can be expressed as the required and adequate requirements of stability:

45 $$s_{1} < 0,\;\;\;s_{1}^{2} - s_{2} s_{3} > 0.$$

## The frequency–response curves and numerical validations

This section investigates the operational characteristics of the model under consideration by resolving the system frequency–response equations at the fundamental resonance situation^35^. The assumed outcomes are attained based on the selection of the dimensionless system data as: $$\omega = 4.5,\,\mu = 0.05,\,\alpha = 0.25,\,\beta = 0.5,$$$$F = 0.1,$$$$\gamma = 0,\,\lambda = 0,$$ and $$\tau = 0$$. Furthermore, the numerical approach to the used parameters yields different figures explaining the structure of the fundamental equation Eq. (1) with the help of the Mathematica Software, at various amounts of the equivalent parameters $$\sigma ,F,\gamma ,\lambda ,$$ and $$\tau$$. The stationary-situation of the vibration amplitude is numerically attained. The next subsections are structured so that subsection "Unrestrained structure" can examine the oscillatory behaviors of the unrestrained scheme, and subsection "The controlled system without time- delay" compares the effectiveness of the two suggested controls at reducing vibrations while ignoring loop delay. The final part "The controlled system with time-delay" investigates how the two controls behave dynamically when the loop delay is reflected.

### Unrestrained Structure

**Figure 7:**
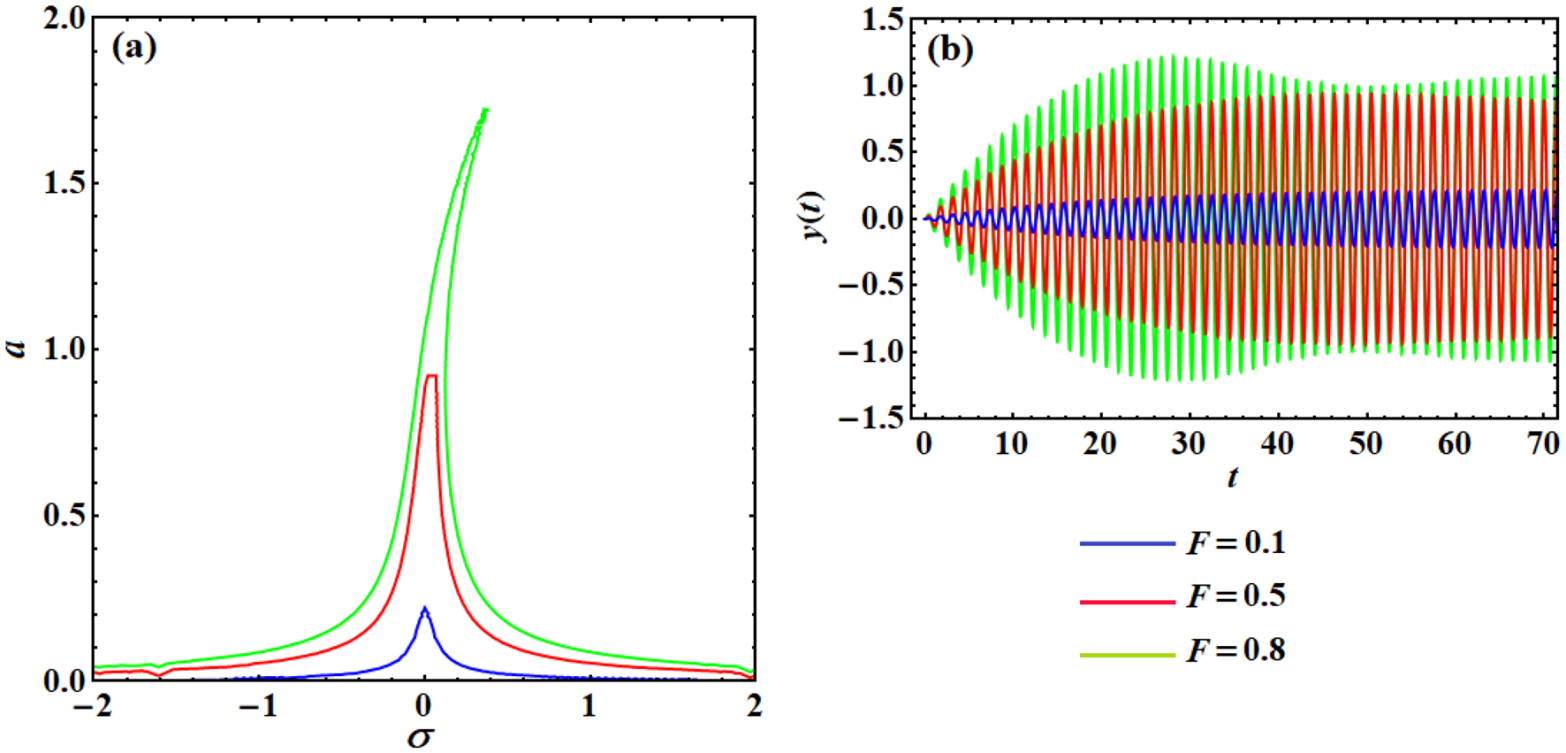
(**a**) Represents the frequency–response’s curves of the uncontrolled system at $F( = 0.1,0.5,0.8)$, while (**b**) shows the time histories of the system according to plotted curves of (**a**) at $\sigma = 0$ for the same values of $F$.

The nearness of the excitement frequency $\Omega$ to the structure natural frequency $\omega$ is formulated using a detuning factor $\sigma$. Consequently, one may employ $\sigma$ as a controller factor to examine the system frequency–response curve in the main resonance situation. Figure 7a examines the frequency–response curves of the uncontrolled system at various stages of the excitement strength $F$. The plotted curves in this figure illustrate that the vibrations of the system monotonically increase with the increase of this force. Furthermore, the scheme may be excited with the amplitude of the considerable oscillation, which depends on both the excitement frequency $\Omega$ and the excitement amplitude $F$. On the other hand, curves of Fig. 7b represent the numerical simulation of the oscillations time histories which corresponds to the curves in Fig. 7a at $\sigma = 0$ (i.e., when $\Omega = \omega$). The inspection of both figures shows that the comparison between them has the same increasing behavior of the amplitude with the increase of $F$ values.

### The controlled system without time- delay

**Figure 8:**
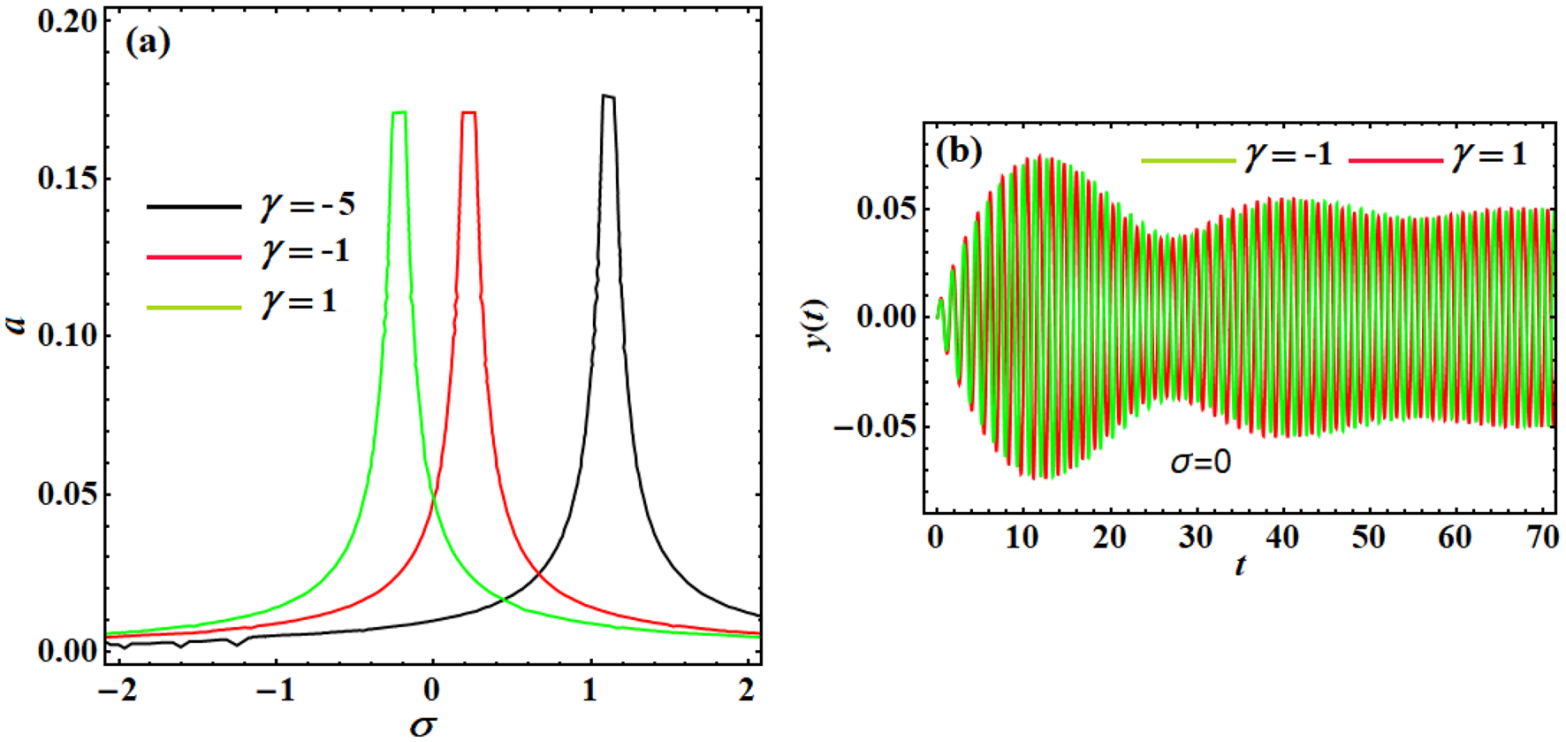
(**a**) Describes the frequency–response curves of the controlled scheme at various values of the position gain $\gamma ( = 1, - 1, - 5)$ when $\lambda = \tau = 0,F = 1$, and (**b**) explores the system’s temporary response when $\gamma ( = - 1,1)$ at $\sigma = 0,F = 0.1$ (i.e., the linear-position control is just stimulated).

As shown in the following discussion, the impacts of the control factors $\gamma$ and $\lambda$ on the scheme response curves are plotted in Fig. 8. Figure 8a demonstrates the influence of the situation gain $\gamma$ on the frequency–response curves of the system. The linear controller hypothesis makes it clear that the position of the feedback controller is what causes changes to the linear natural frequency system. Accordingly, the frequency–response curve shifts as the positional gains grow.

In this subsection, the effect of the two controls gains on the oscillatory behaviors of the scheme is examined when the loop delay vanishes. It is clear from Fig. 8a that**,** based on $\gamma$ values, the linear-position controller is accountable for any variation of the scheme frequency response curve. Consequently, it is preferable to ignore the amplitude high oscillation of the system with the variation of its normal frequency, away from the excitement frequency, by using the linear position. The numerical reproductions, for the controlled scheme temporal vibrations according to the plotted curves in Fig. 8a at $\sigma = 0$ (i.e., $\Omega = \omega$), are graphed in Fig. 8b. The numerical solutions of the original Eq. (1) are in high agreement with the behaviors of the curves included in Fig. 8a. Moreover, the oscillations of Fig. 8a have some convergent beams and a stable manner to the end of the examined time interval.

**Figure 9:**
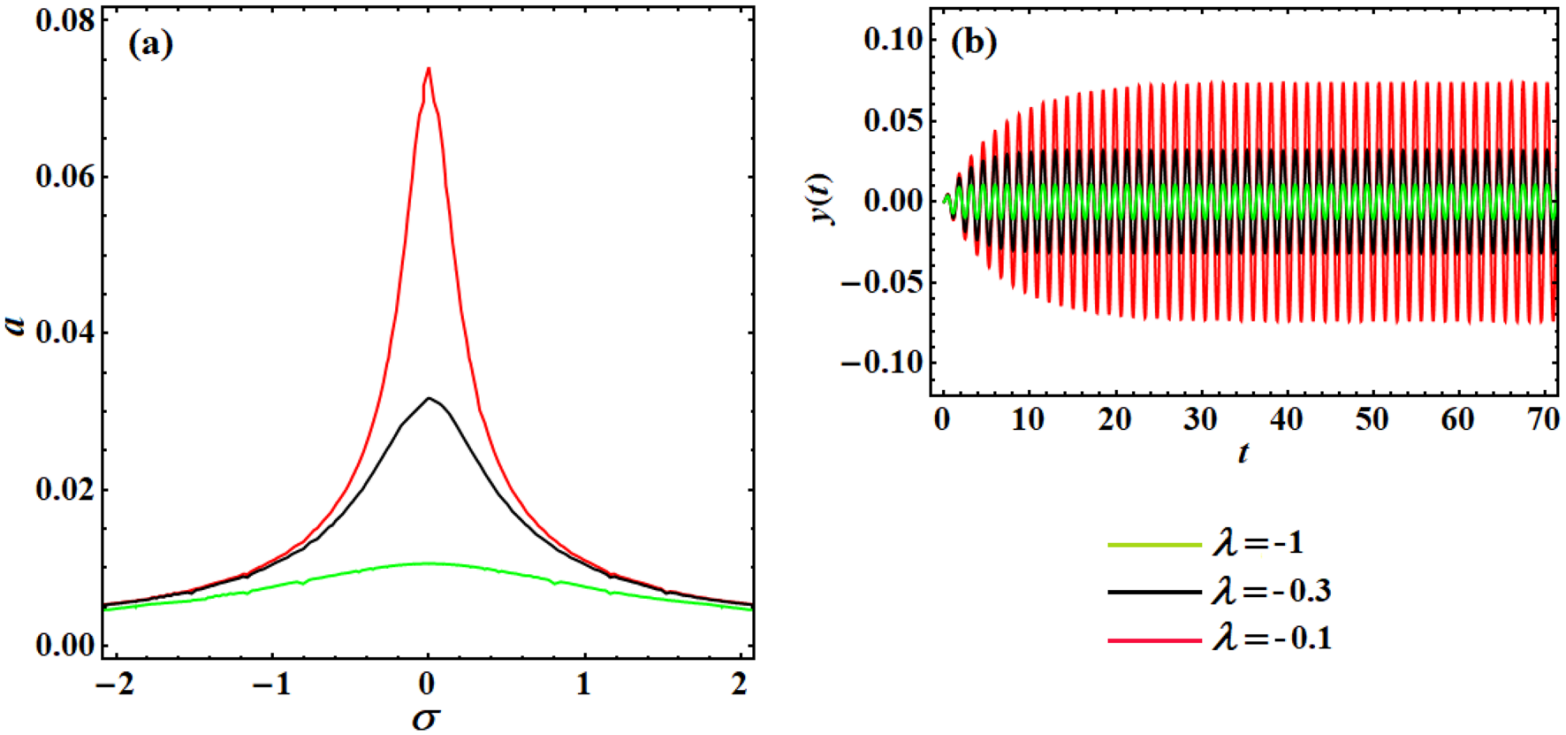
Illustrates the effect of positive linear-velocity feedback gain of the system frequency–response curve when the loop-delay is zero: (**a**) shows the impact of the linear-velocity gain $\lambda$, while (**b**) reveals the system time response when $\lambda ( = - 1, - 0.3, - 0.1)$.

The curves in Fig. 9a show the influence of decreasing the linear-velocity feedback gain $\lambda$ on the scheme frequency–response curve, where the decrease of $\lambda$ improves the structure of the linear damping parameter, which eventually decreases the scheme vibration amplitudes. It is observed that with the increase of $\lambda$, the frequency response curve increases, where three pick points are observed with the used values of $\lambda$. The nonlinear temporal vibration of the beam system, when decreasing the velocity feedback gain from $\lambda = - 0.1$ to $\lambda = - 1$ according to Fig. 9a, is numerically proven in Fig. 9b. The comparison between the behaviors of both figures reveals good consistency between their behaviors.

### The controlled system with time-delay

**Figure 10:**
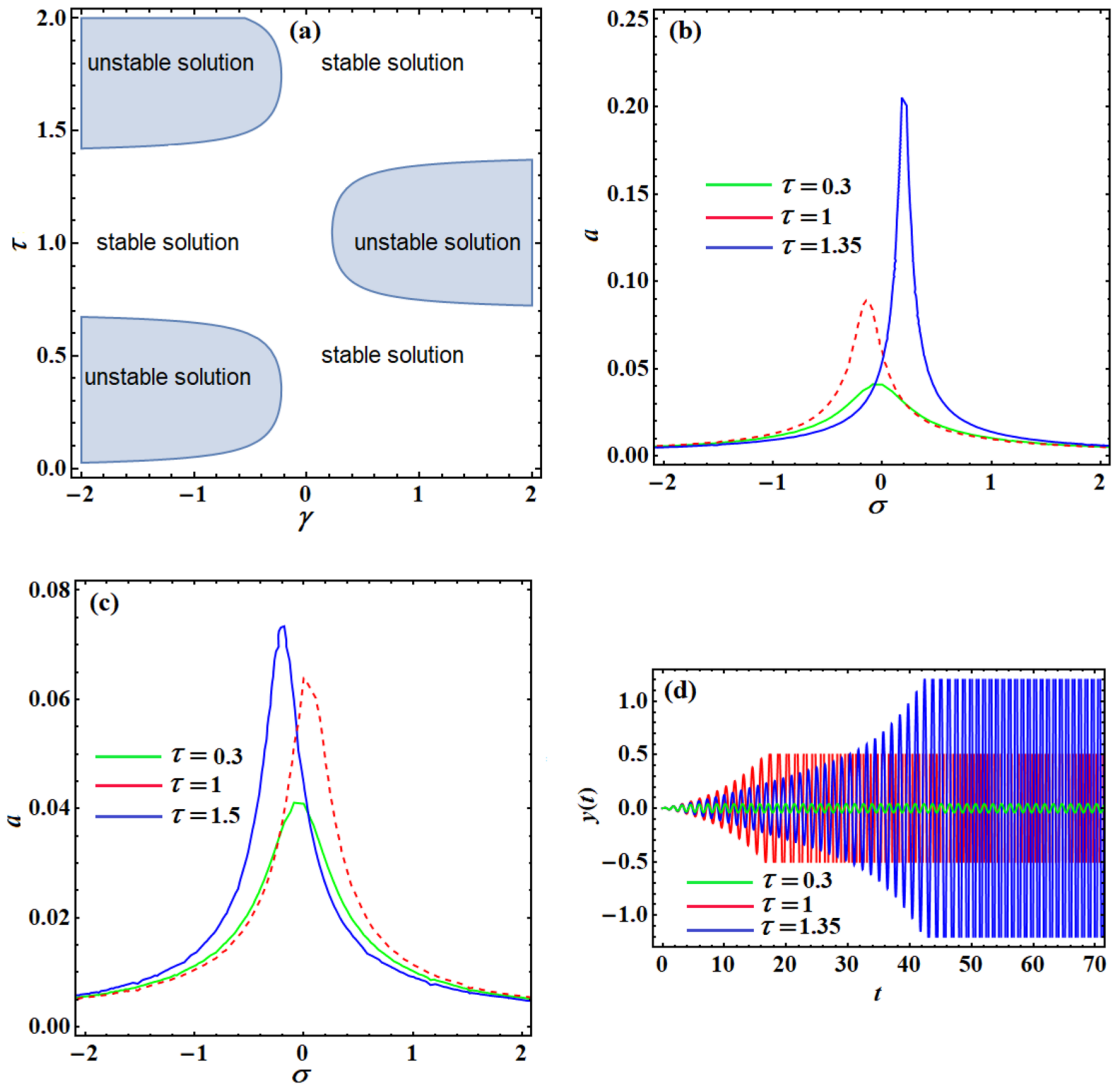
(**a**) Represents the stable and unstable solution zone in $(\gamma - \tau )$ plane at $F = 0.1,\,\,\sigma = \lambda = 0$, (**b**) displays the system frequency–response curves when $\gamma = - 1,F = 0.1,\,\lambda = 0$ at different time-delay values, (**c**) reveals the system frequency–response curve when $\gamma = 1,F = 0.1,\,\lambda = 0$ at various values of time-delay parameter, and Fig. 10d describes the time response of system when $\gamma ( = - 1,1)$.

To discover the impact of the controller loop-delay $\tau$ on the stability and the vibration amplitude of the system, curves of Fig. 10a are plotted. The position gain $\gamma$ is employed as a controller factor against the time delay $\tau$ whenever $\sigma = \lambda = 0$ to describe the stable and unstable zones in $(\gamma - \tau )$ plane. This figure demonstrates that the unstable zone of the solution is a periodic function of time-delay either for negative or positive position gain. Consequently, it is likely to choose the loop delay in a particular approach for a positive or negative position gain to ensure the stability of the system. Figure 10b,c display the system frequency–response curve at various amounts of $\gamma$ and $\tau$ which have been selected according to Fig. 10a. Figure 10b expresses the frequency–response curves of the examined scheme at three various values of $\tau$ when $\gamma = - 1$ (for the case of negative position feedback). The examination of this figure shows that the smallest vibration stage happens when $\tau = 0.3$, while the scheme oscillates at a great vibration amplitude when $\tau = 1.35$. Figure 10c reveals the frequency–response curves at different values of $\tau$ for the case of a positive position feedback $\gamma = 1$, which ensures that the lowest vibration stage and the large vibration amplitudes response occur at $\tau = 0.3$ and $\tau = 1.5,$ respectively. Consequently, one can improve the vibration decreasing efficiency of the control employing the loop-delays as a new controller factor, where the optimal amounts of the loop-delay $\tau$ that can improve the control efficiency must be chosen within the middle of the stable solution zone. At $\tau ( = 0.3,1)$, the system frequency–response curve has two unstable regions in $(\gamma - \tau )$ plane at $\gamma ( = - 1,1)$, where the structure will achieve unstable motion for any excitation frequency. To confirm the precision of the achieved stability chart in Fig. 10a, a numerical imitation for the deemed scheme has been graphed in Fig. 10d. The comparison of the curves included in Fig. 10b with Fig. 10d displays that the structure replies with bounded oscillation amplitude as $\tau ( = 1,1.35)$ because these points are chosen inside the stable zone of the solution. As opposed to that, the regulated scheme replies with boundless oscillation amplitude at $\tau = 0.5$ as this value has been chosen inside the unstable solution zone. The comparison between Figs. 10c,d has the same direction as before with Figs. 10b,d.

**Figure 11:**
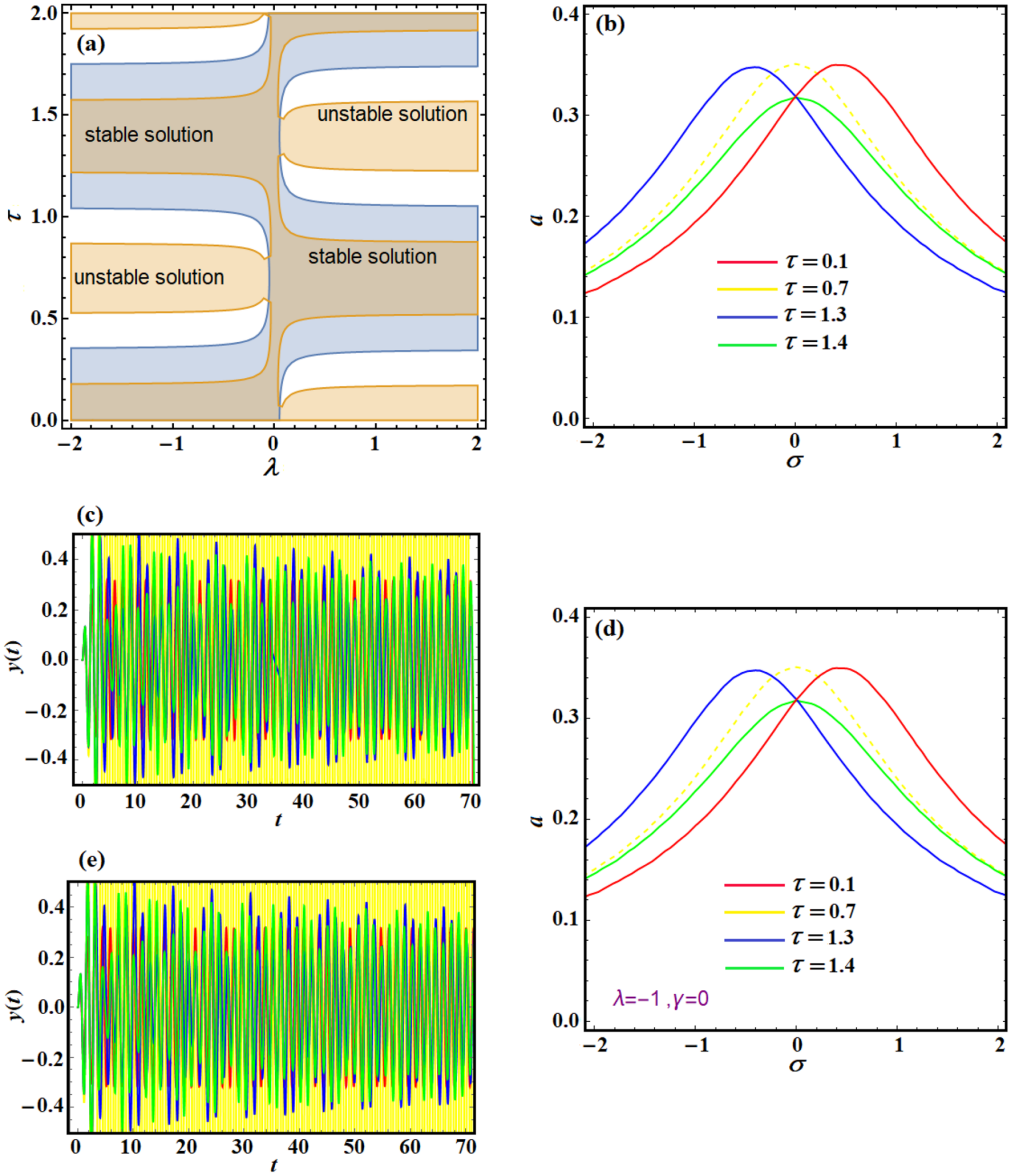
(**a**) Shows the stable and unstable solution zone in $(\lambda - \tau )$ plane when $\sigma = \gamma = 0$, (**b**) describes the frequency–response curves of the system at $\lambda = - 0.1,\gamma = 0,F = 3$ for different values of the time-delay parameter, (**c**) explores the system time response when $\tau ( = 0.1,0.7,1.3,1.4)$, (**d**) reveals the system frequency–response curves at $\lambda = 0.2,\gamma = 0,F = 2.5$ for various values of time-delay parameter, and (**e**) shows the system time response at $\tau ( = 0.1,0.7,1.3,1.4)$.

The plotted zones in Fig. 11a illustrate the stable and unstable solution areas in the plane $(\lambda - \tau )$ when $\sigma = \gamma = 0$. The graphs demonstrate that these zones are periodic functions of either positive or negative velocity gain in terms of time-delay. The structure frequency–response curve at various points in $(\lambda - \tau )$ plane is depicted in Fig. 11b–e. Furthermore, the curves of Fig. 11b describe the frequency–response curve of the scheme and are computed when the velocity feedback gain $\lambda = - 1$ at $\tau ( = 0.1,0.7,1.3,1.4)$ to show the influence of the loop-delay on the scheme, where the best time-delay at speed feedback gain is found at $\tau = 1.4.$

The three values of delay $\tau ( = 0.1,1.3,1.4)$ have the same amplitude at $\sigma = 0$, which is numerically in an agreement with Fig. 11c. The controlled scheme replies with a boundless oscillation amplitude at $\tau = 0.7$ and it is of the same consistency with the corresponding curve behavior of Fig. 11c at the same value of $\tau$. When $\tau ( = 0.1,0.7,1.3,1.4)$ the scheme frequency–response curve of speed feedback gain $\lambda = 0.2$ is demonstrated in Fig. 11d, where the best time-delay for vibration destruction is at $\tau = 0.7$. Based on Fig. 11a, choosing the loop-delay to be at the center of the stable solution zone in $(\lambda - \tau )$ plane improves the effectiveness of the controller. Moreover, Fig. 11d has two stable and two unstable zones in the plane $(\lambda - \tau )$ at $\lambda ( = 0.2, - 0.2)$ and $\tau ( = 0.7,1.3)$, where the structure will operate unstable motion for any excitation frequency. Figure 11d shows high consistency with Fig. 11e.

**Figure 12:**
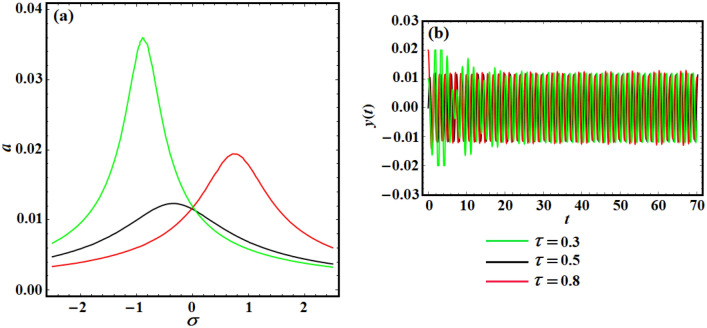
(**a**) Represents the system frequency–response curves at $\gamma = 2,\;\lambda = 0.8,\;F = 0.1$ for various amounts of the time-delay parameter, and (**b**) shows the system time response, when $\gamma = 2,\lambda = 0.8$.

It is likely to prevent the structure high oscillation amplitude with the variation of the linear-position and the linear-velocity feedback control. As shown in Fig. 12a, the linear-position with linear-velocity feedback control is more effective with time delay in reducing the structure nonlinear vibrations. It is described that the optimal time-delays amounts can increase the structure strength against instability based on the obtained analytical and numerical investigations in Fig. 12b. Furthermore, the system vibrations should be chosen so that they maximize the linear damping function $\mu_{Eq} = \mu + ({\gamma \mathord{\left/ {\vphantom {\gamma \omega }} \right. \kern-0pt} \omega })\sin (\omega \tau ) - \lambda \cos (\omega \tau )$ to increase the linear (position-velocity) controllers efficiency in the suppression case. Additionally, it is found that the change in time delay suppresses the system vibration. Based on the curves of Fig. 12a, we can verify that the curves in Fig. 12b represent the optimal control strategy for reducing the extreme oscillation amplitude of a parametrically excited structure as well as feedback gains (i.e., $\gamma = 2,\lambda = 0.8$).

## Conclusions

The subject of this paper is the time-delayed controllers of a damped nonlinear stimulated DO. Since time-delayed techniques have lately attracted a lot of research attention, the investigation subject is very contemporary. Time delays of position and velocity are used to reduce the nonlinear oscillation of the existing model. A modified HPM is employed to obtain a significantly more precise approximate solution^36–38^. For various amounts of the used factors, the temporal difference of this solution is graphed. The analytical approximate solution has been compared with the numerical solution, which has been obtained by employing the RK4 and they have proved consistent. The results have demonstrated that the improved HPM is suitable for a range of damped nonlinear oscillators because it reduces solution error while boosting validation diversity. Additionally, it offers a viable model that addresses a range of nonlinear issues. Gained has been the nonlinear algebraic equation that controls the steady-state oscillation amplitude. Investigations have been done on the effectiveness of the suggested controller, the influence of time delays, control gains, and feedback gains. The actual results have demonstrated how the sum of the time delays in the control loop and the product of the control and feedback gains affect the controller performance. Analytical and numerical analyses have shown that optimizing the suggested controller could entirely cut down on system vibrations for particular values of the control and feedback signal. It is possible to obtain the nonlinear algebraic equation governing the steady-state oscillation amplitude. When there are time delays as well as control and feedback gains, the controller performs well. Though the method adopted in this study is traditional, it has yielded novel results. The findings have shown that the sum of time delays in the control loop as well as the product of control and feedback gains have an effect on the controller effectiveness. Theoretical and numerical models have demonstrated that the suggested control may successfully lessen structure vibrations at suitable levels of the controller and feedback signal gains.

## Data Availability

Since no datasets were accumulated or handled throughout the existing work, data sharing was not appropriate to this paper.

## List of symbols

$y$: Displacement from the equilibrium position.
$$t$$: Proper time.
$^{.}$: Dot signifies the derivative of $y$ with regard to $t$.
$\omega$: Root of the natural frequency of the scheme.
$\mu$: Pure linear damping coefficient.
$\alpha$: Cubic nonlinear Duffing coefficient, $\alpha > 0$, and $\alpha < 0$ are hardening and softening spring, respectively.
$\beta$: Quintic nonlinear Duffing coefficient.
$$F$$: Force’s amplitude.
$$\Omega$$: Force’s frequency.
$\gamma$: Positive position feedback controller.
$\lambda$: Positive velocity feedback controller.
$A$: Initial amplitude parameter.
$\tau$: Time-decay control parameter.
$\rho$: Small artificial parameters.
$F_{c}$: The control force
